# The Efficacy and Safety of Intranasal Corticosteroids in Chronic Rhinosinusitis: A Systematic Review

**DOI:** 10.7759/cureus.87674

**Published:** 2025-07-10

**Authors:** Abdualllah A Mawkili, Jarallah H Alghazi, Abdullah M Alqahtani, Mohammed A Alshalwan, Shahd Alallah M Essa, Ghadah I Alissa, Abdulrazaq I Althobaiti, Rakan Y Alsuwayyid, Saeed E Alzahrani, Saud T Alaidarous, Ahmed Y Ayoub

**Affiliations:** 1 Surgery, Jazan University, Jazan, SAU; 2 College of Medicine, King Saud bin Abdulaziz for Health Sciences, Riyadh, SAU; 3 College of Medicine, King Saud bin Abdulaziz University for Health Sciences, Riyadh, SAU; 4 College of Medicine, King Khalid University, Abha, SAU; 5 College of Medicine, Jazan University, Jazan, SAU; 6 College of Medicine, Sulaiman AlRajhi University, Al Qassim, SAU; 7 College of Medicine, Ibn Sina National College for Medical Studies, Jeddah, SAU; 8 College of Medicine, Majmaah University, Almajmaah, SAU; 9 College of Medicine, Umm Al-Qura University, Makkah, SAU

**Keywords:** biologics, chronic rhinosinusitis, crssnp, crswnp, dupilumab, exhalation delivery system, fluticasone, intranasal corticosteroids, mepolizumab, nasal polyps

## Abstract

Chronic rhinosinusitis (CRS), with or without nasal polyps (CRSwNP/CRSsNP), is a prevalent inflammatory condition of the sinonasal mucosa, for which intranasal corticosteroids (INCS) are widely used as first-line therapy. This systematic review evaluated the efficacy and safety of INCS, used alone or with adjunctive treatments, in improving clinical outcomes in CRS patients. A comprehensive literature search was conducted across PubMed, Cochrane Library, Scopus, Web of Science, and the Virtual Health Library (VHL) through May 2025, including randomized controlled trials (RCTs) assessing INCS efficacy in adults and children with CRS. Outcomes examined included nasal polyp score (NPS), nasal congestion, quality of life (QoL), sinus opacification, olfactory function, acute exacerbations, and adverse events.

Six high-quality RCTs involving 2,339 participants were included. Dupilumab demonstrated the greatest NPS reduction (Δ−2.06), improved nasal congestion, and reduced the need for surgery or systemic steroids. Mepolizumab, omalizumab, and benralizumab were also effective in reducing polyp size and symptoms, particularly in eosinophilic CRS. EDS-FLU (exhalation delivery system with fluticasone) improved congestion, SNOT-22 (22-item Sino-Nasal Outcome Test) scores, and reduced acute exacerbations. In pediatric patients, mometasone delivered with saline nebulization significantly improved SN-5 scores. All interventions were well tolerated, with few adverse events reported. Overall, INCS are effective in managing CRS symptoms and reducing disease burden, particularly when combined with biologics or advanced delivery systems, and remain a cornerstone of CRS management with a strong safety and efficacy profile across diverse populations.

## Introduction and background

Chronic rhinosinusitis (CRS) is a persistent inflammatory condition of the nasal and paranasal sinus mucosa that significantly impacts quality of life and healthcare systems worldwide. It is commonly classified into CRS with nasal polyps (CRSwNP) and without nasal polyps (CRSsNP), based on endoscopic and histologic findings [1]. The global prevalence of CRS is estimated at 10-12%, with variation across geographic and demographic populations [2]. Symptoms such as nasal obstruction, facial pressure, and hyposmia often persist for over 12 weeks and are frequently underdiagnosed or mismanaged, especially in primary care settings. CRS has a complex, multifactorial pathophysiology involving environmental triggers, microbial agents, and dysregulated immune responses. Eosinophilic inflammation, especially in CRSwNP, is linked to more severe disease and higher recurrence rates after treatment [3], whereas CRSsNP often features neutrophilic inflammation and responds less favorably to corticosteroids [4]. This heterogeneity underscores the need for personalized anti-inflammatory strategies.

Intranasal corticosteroids (INCS) are the cornerstone of medical management in both CRSwNP and CRSsNP, acting locally to reduce mucosal inflammation and alleviate symptoms. They are widely recommended as first-line pharmacologic therapy due to their proven efficacy and favorable safety profile [5]. However, treatment responses can vary, and effectiveness may be limited by factors such as poor delivery in cases of severe nasal obstruction [6]. To address these limitations, novel delivery systems-such as exhalation devices, corticosteroid irrigations, and combination regimens with systemic or biologic agents-have been developed to enhance drug distribution and reach deeper sinonasal regions [7]. Despite promising outcomes, inconsistencies in study design and outcome measures have made it difficult to compare the relative benefits of these approaches across CRS subtypes [8].

Randomized controlled trials (RCTs) and meta-analyses consistently support the use of INCS to reduce polyp size, improve nasal airflow, and delay the need for surgery, particularly in CRSwNP [9]. However, their role in CRSsNP and pediatric CRS remains less clear, and factors like comorbid asthma or aspirin-exacerbated respiratory disease (AERD) may influence treatment outcomes [10]. Given the clinical variability and methodological diversity across studies, a comprehensive synthesis of high-quality evidence is needed. This systematic review aims to evaluate the efficacy of intranasal corticosteroids in CRSwNP and CRSsNP, based on RCTs assessing symptom control, polyp regression, radiologic changes, and quality of life.

## Review

Methods

Search Strategy and Registration

This systematic review was registered with PROSPERO, the International Prospective Register of Systematic Reviews. It was conducted and reported according to the Preferred Reporting Items for Systematic Reviews and Meta-Analyses (PRISMA) guidelines. A comprehensive literature search was performed across PubMed, Cochrane Library (CENTRAL), Scopus, Web of Science, and the Virtual Health Library (VHL), covering all records up to May 20, 2025. A combination of controlled vocabulary and free-text terms was used, including: “chronic rhinosinusitis,” “CRS,” “nasal polyps,” “CRSwNP,” “CRSsNP,” “intranasal corticosteroids,” “fluticasone,” “mometasone,” “budesonide,” “beclomethasone,” and “topical steroids.” Search strategies were adapted to each database using appropriate Boolean operators. Filters were applied to restrict results to English-language human studies. Reference lists of included articles were also manually screened to identify additional eligible studies.

Eligibility Criteria

Eligibility criteria were established using the PICO (Population, Intervention, Comparison, and Outcome) framework. Included studies were RCTs published in English that (1) involved adult or pediatric patients with CRS with or without nasal polyps; (2) evaluated INCS as monotherapy or in combination with other treatments; (3) compared interventions with placebo, standard care, or other active comparators; and (4) reported at least one clinical or patient-reported outcome, such as nasal polyp score (NPS), symptom severity, imaging findings, or quality of life. Case reports, reviews, editorials, trial registrations, animal studies, non-English publications, conference abstracts, and studies with non-comparative observational designs were excluded.

Study Selection

Two reviewers independently screened titles and abstracts based on the predefined criteria. Full texts were retrieved for studies that met the inclusion criteria or had insufficient information in the abstract. Discrepancies were resolved through discussion, and if necessary, adjudicated by a third reviewer.

Data Extraction

Data from the included studies were extracted using a standardized form. Extracted information included study design, country of origin, sample size, population characteristics, type and dosage of INCS, route and duration of administration, comparator interventions, outcome measures [e.g., NPS, nasal congestion, SNOT-22 (22-item Sino-Nasal Outcome Test) scores, CT findings], and primary results. Any disagreements were resolved through discussion to ensure accuracy and completeness.

Quality Appraisal

The methodological quality of each study was independently assessed by two reviewers using the modified Downs and Black checklist. This tool evaluates four domains: reporting, external validity, internal validity (bias and confounding), and statistical power. Total scores range from 0 to 28, with studies rated as excellent (26-28), good (20-25), fair (15-19), or poor (≤14). Discrepancies in scoring were resolved by consensus or adjudicated by a third reviewer when needed.

Results

Study Selection

**Figure 1:**
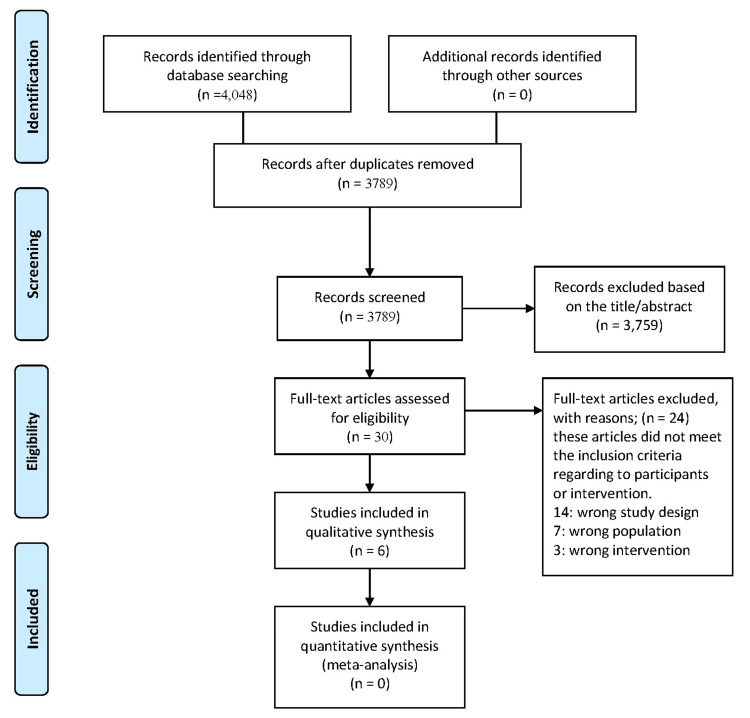
PRISMA flow diagram illustrating the study selection process for the systematic review on intranasal corticosteroids in chronic rhinosinusitis PRISMA: Preferred Reporting Items for Systematic Reviews and Meta-Analyses

A total of 4,048 records were identified through database searches: PubMed (173), Cochrane Library (585), Scopus (1,678), Web of Science (1,347), VHL (265). After removing 259 duplicates, 3,789 records were screened by title and abstract. Of these, 3,759 were excluded as irrelevant, and 30 full-text articles were assessed. Twenty-four were excluded due to incorrect study design (n=14), population (n=7), or intervention (n=3). Ultimately, six studies were included in the qualitative synthesis [11-16]. No meta-analysis was conducted due to significant heterogeneity in interventions, populations, and outcomes (Figure 1).

Study Characteristics

The six included RCTs varied in design, population, and treatment strategy. Five focused on adult CRSwNP, while one (Latek et al. [11]) addressed pediatric CRS. Palmer et al. [15] was the only study to include both CRSwNP and CRSsNP. Sample sizes ranged from 63 (Latek et al. [11]) to 724 (Bachert et al. [13]). Most studies investigated biologics (dupilumab, mepolizumab, omalizumab, benralizumab) as add-ons to standard INCS; Palmer et al. [15] evaluated a device-enhanced delivery system (EDS-FLU). Common outcomes included NPS, congestion, sinus opacification, quality of life (e.g., SNOT-22, SN-5), and need for systemic therapy or surgery. Overall, all interventions were well tolerated and demonstrated varying degrees of clinical benefit (Table 1).

**Table 1:** Summary of included RCTs evaluating INCS in CRS CRS: chronic rhinosinusitis; CRSwNP: CRS with nasal polyps; CRSsNP: CRS without nasal polyps; RCT: randomized controlled trial; INCS: intranasal corticosteroid; SC: subcutaneous; q2w/q4w/q8w: every 2/4/8 weeks; SoC: standard of care; EDS-FLU: exhalation delivery system with fluticasone; AERD/NSAID-ERD: aspirin-exacerbated or NSAID-exacerbated respiratory disease; NPS: nasal polyp score; NBS: nasal blockage score; VAS: visual analog scale; SNOT-22: 22-item Sino-Nasal Outcome Test; CSS: composite symptom score; PGIC: Patient Global Impression of Change; LMS: Lund-Mackay score (CT); UPSIT: University of Pennsylvania Smell Identification Test; TNSS: total nasal symptom score; ILC3: type 3 innate lymphoid cells; OTU: operational taxonomic unit (microbial richness)

Study ID	Study design	Country/region	Study characteristics	Population characteristics	Intervention details	Outcome measures	Results
Latek et al., 2023 [11]	Open-label randomized clinical trial	Poland	N=63 children; 12-week intervention; mometasone + saline nebulizer vs saline alone	Children 4–8 years with CRS (EPOS 2012); 66.7% had asthma; excluded tonsillar hypertrophy or immunodeficiency	Mometasone furoate (1 spray/nostril daily) + saline nebulizer vs saline nebulizer alone	SN-5 QoL score, microbiome diversity (OTU richness), nasal ILC3 abundance	Significant SN-5 reduction (p=0.009); increased OTU richness (p=0.04); reduced ILC3 (p=0.03); no adverse events
Bachert et al., 2022 (benralizumab) [12]	Phase III, randomized, double-blind, placebo-controlled (OSTRO)	Multinational (Europe & USA)	N=413; 40–56 weeks; benralizumab vs placebo + mometasone	Adults 18–75 with CRSwNP; 67.8% asthma, 73.2% prior NP surgery, mean eosinophils ~450	Benralizumab 30 mg SC q4w ×3, then q8w + mometasone vs placebo + mometasone	NPS, Nasal Blockage Score, SNOT-22, DSS, LMS, UPSIT	NPS Δ−0.57 (p<0.001); NBS Δ−0.27 (p=0.005); 35.3% had ≥1-point NPS improvement vs 19.2% in placebo
Bachert et al., 2019 [13]	Two multicentre, randomized, double-blind, placebo-controlled phase 3 trials (SINUS-24 and SINUS-52)	Multinational (27 countries)	N=724; SINUS-24: 24 weeks, SINUS-52: 52 weeks; dupilumab vs placebo; all received mometasone	Adults ≥18 with CRSwNP; 59% had asthma, 28% had NSAID-ERD	Dupilumab 300 mg SC q2w + daily mometasone vs placebo + mometasone	NPS, Nasal Congestion, CT Lund-Mackay, SNOT-22, UPSIT, need for surgery/systemic steroids	Significant improvement in NPS (Δ−2.06), congestion, and all secondary outcomes; reduced need for surgery/steroids
Gevaert et al., 2020 [14]	Two phase III RCTs (POLYP 1 & 2)	Multinational (North America & Europe)	N=265; 24 weeks; omalizumab vs placebo + mometasone	Adults with CRSwNP unresponsive to INCS; ~50–60% with asthma, ~27% NSAID-ERD, 60% with prior surgery	Omalizumab SC q2w/q4w (75–600 mg based on IgE) + mometasone vs placebo + mometasone	NPS, Nasal Congestion, SNOT-22, UPSIT, TNSS, need for surgery	Improved NPS (−1.14 vs 0.06), SNOT-22, smell; reduced systemic steroid use; well tolerated
Palmer et al., 2024 [15]	Two phase III RCTs (ReOpen1 & ReOpen2)	Multinational (13 countries)	N=555; 24 weeks; ReOpen1 (CRS ± polyps), ReOpen2 (CRSsNP); EDS-FLU vs EDS-placebo	Adults ≥18 with CRS diagnosed via symptoms and CT; ~60% prior INCS use, ~40% prior surgery; ReOpen2 excluded polyps	EDS-FLU (186 or 372 mcg BID) vs EDS-placebo for 24 weeks	CSS, CT sinus opacification, acute exacerbations, SNOT-22, PGIC	Significant improvement in CSS and CT score; reduced exacerbations (56–66%); SNOT-22 improvement; similar benefit in prior INCS users
Bachert et al., 2022 (Mepolizumab) [16]	Phase III, randomized, double-blind, placebo-controlled (SYNAPSE)	Multinational	N=407; 52 weeks; mepolizumab vs placebo + SoC; all received mometasone	Adults with severe CRSwNP; ≥1 prior surgery; 71% asthma, 26.5% NSAID-ERD; subgroups by eosinophils	Mepolizumab 100 mg SC q4w vs placebo + SoC (INCS, saline, etc.)	NPS, VAS obstruction, SNOT-22, surgery/systemic steroids, UPSIT	Greater improvement in NPS (50.5% vs 28.4%), VAS (60.2% vs 36.3%), reduced surgery and steroid use; stronger effect in the eosinophilic subgroup

Risk of Bias and Quality Assessment

All six studies demonstrated high methodological quality based on the Downs and Black checklist. Reporting scores were near-perfect in five studies. External validity was rated 2/3 due to strict eligibility criteria. Internal validity related to bias scored consistently high (6/7), with effective blinding and outcome measures. Confounding and power domains were also well addressed. Only the pediatric study by Latek et al. [11] scored slightly lower (4/6) in confounding, likely due to its smaller sample and open-label design. Overall, quality scores ranged from 22 to 24 out of 27, indicating strong methodological rigor (Table 2).

**Table 2:** Quality assessment of included studies using the Downs and Black checklist This table summarizes the methodological quality scores for the six included randomized controlled trials assessing intranasal corticosteroids in chronic rhinosinusitis. The Modified Downs and Black checklist, a 27-item tool, was used to evaluate study quality across five domains: reporting (0–10), external validity (0–3), internal validity—bias (0–7), internal validity—confounding (0–6), and statistical power (0–1). Total scores range from 0 to 27, with higher scores indicating better study quality

Study	Reporting (0–10)	External validity (0–3)	Internal validity – bias (0–7)	Internal validity – confounding (0–6)	Power (0–1)	Total score (0–27)
Latek et al., 2023 [10]	9	2	6	4	1	22
Bachert et al., 2022 (benralizumab) [11]	10	2	6	5	1	24
Bachert et al., 2019 [12]	10	2	6	5	1	24
Gevaert et al., 2020 [13]	10	2	6	5	1	24
Palmer et al., 2024 [14]	10	2	6	5	1	24
Bachert et al., 2022 (Mepolizumab) [15]	10	2	6	5	1	24

Nasal Polyp Score

Five studies assessed NPS. Dupilumab produced the largest reductions: −2.06 and −1.80 in SINUS-24 and SINUS-52, respectively (p<0.0001) [13]. Mepolizumab also showed strong results, with ≥1-point reductions in 50.5% of patients vs 28.4% in placebo (p<0.0001) [16]. Omalizumab reduced NPS by −1.14 and −0.59 in POLYP 1 and 2 (p<0.0001; p=0.0140) [14]. Benralizumab had a modest reduction of −0.57 at week 40 (p<0.001) [16]. Palmer et al. [15] did not measure NPS but reported significant reductions in sinus opacification. Overall, biologic-enhanced INCS therapy yielded the greatest improvements in polyp burden.

Nasal Congestion or Obstruction

All six studies assessed congestion. Dupilumab improved scores by −0.89 to −0.98 vs placebo (p<0.0001) [13]. Mepolizumab led to ≥3-point VAS improvements in 60.2% vs 36.3% (p<0.0001) [16]. Omalizumab showed reductions of −0.55 and −0.50 (p=0.0004; p=0.0017) [14]. Benralizumab improved congestion by −0.27 (p=0.005) [12]. Palmer et al. [15] reported rapid symptom relief with EDS-FLU (Δ-1.62 vs −0.70; p<0.001). These consistent findings support INCS efficacy across delivery modes and phenotypes.

Quality of Life

All studies showed improved quality of life. Dupilumab led to SNOT-22 reductions exceeding 20 points (p<0.0001) [13]. Mepolizumab improved by≥9 points in a significantly larger proportion of patients [16]. Omalizumab showed early improvement (Δ-10.43 at week 4) [14]. Benralizumab produced a −7.49 reduction by week 56 (p=0.02) [12]. EDS-FLU also improved SNOT-22 by −10.25 to −12.61 (p<0.001) [15]. In pediatrics, mometasone plus saline reduced SN-5 by −0.58 (p=0.009) [11]. These results confirm the INCS benefit on patient-reported outcomes.

Sinus Opacification

CT-based changes were reported in four studies. Dupilumab significantly reduced Lund-Mackay scores by −7.44 and −5.13 (p<0.0001) [13]. EDS-FLU reduced sinus opacification volume by −5.48 (p<0.001) [15]. Benralizumab showed a non-significant change of −0.99 vs −0.14 [12]. These findings suggest improved anatomic outcomes with INCS, especially with biologic or device enhancements.

Olfactory Function

Smell function improved in five trials. Dupilumab increased UPSIT scores by ~10 points and reduced anosmia from 74% to 24% [13]. Omalizumab showed ~3.5-point gains by week eight [14]. Benralizumab and mepolizumab had limited UPSIT impact but showed subjective improvement [12,16]. Palmer et al. [15] used PGIC ratings, indicating perceived olfactory gains. Dupilumab and omalizumab were most effective for olfactory recovery.

Acute Exacerbations and Rescue Therapy

Five studies evaluated the need for systemic therapy or surgery. Dupilumab reduced the combined rate of surgery or systemic corticosteroids by 74-83% (HR 0.243; p<0.0001) [13]. Mepolizumab reduced surgical risk, especially in eosinophilic patients [16]. Omalizumab lowered rescue corticosteroid use (2.3% vs 6.2%) and surgery need [14]. Benralizumab showed favorable trends, though not statistically significant [12]. EDS-FLU reduced exacerbations by 56-66% (p<0.001) [15]. These data demonstrate that INCS-especially with biologics or optimized delivery-reduce treatment escalation.

Safety and Tolerability

All studies reported good safety profiles. Dupilumab and mepolizumab had fewer adverse events than placebo [13,16]. Omalizumab and benralizumab were well tolerated, with mild AEs most common [12,14]. EDS-FLU was associated with minor local effects (e.g., epistaxis) but no serious events [15]. Latek et al. [11] reported no adverse events in children. Overall, INCS therapies were safe across age groups and CRS phenotypes.

Discussion

This systematic review of six high-quality RCTs confirms that INCS, delivered conventionally, via enhanced systems, or combined with biologics, significantly improve key clinical outcomes in CRS with and without nasal polyps [11-16]. Treatments consistently reduced NPS, congestion, sinus opacification, and improved olfactory function and quality of life. Biologics such as dupilumab, mepolizumab, and omalizumab showed the greatest efficacy, especially in eosinophilic CRS, while device-assisted delivery systems like EDS-FLU enhanced symptom control by improving drug deposition [12-16]. Reductions in the need for systemic corticosteroids and surgery were also observed, highlighting the potential of these therapies to reduce disease burden and treatment escalation [11-16].

All interventions were well tolerated, with low rates of adverse events reported across diverse patient populations, including pediatric cases [11-16]. This review supports INCS as a cornerstone of CRS management, with biologics and novel delivery methods offering important advancements that optimize treatment outcomes. Together, these findings underscore the importance of personalized approaches to CRS therapy, tailoring treatments based on phenotype, severity, and patient needs to maximize efficacy and safety

Limitations and Future Directions

Limitations of this review include restriction to English-language articles and exclusion of observational or real-world effectiveness studies, which may reduce generalizability. Heterogeneity in outcome measures, treatment durations, and patient phenotypes (e.g., eosinophilic vs neutrophilic CRS) also complicates direct comparisons. Additionally, limited pediatric data constrain applicability to younger populations.

Future research should focus on head-to-head trials comparing different INCS molecules and delivery systems, especially in CRSsNP, where treatment response is often suboptimal. Studies are also needed on long-term adherence, real-world effectiveness, and outcomes in understudied populations such as children and patients with multiple comorbidities. Biomarker-guided treatment stratification may further personalize INCS therapy and improve clinical outcomes.

## Conclusions

Findings of this systematic review confirm that INCS are both effective and safe for managing CRS, particularly in patients with nasal polyps. INCS therapies consistently produce significant improvements in nasal polyp size, nasal congestion, olfactory function, and overall quality of life, while also reducing the reliance on systemic corticosteroids and the need for surgical interventions. The addition of biologic agents and advanced delivery systems further enhances these benefits, offering promising avenues for more personalized and effective treatment strategies. Moving forward, optimizing delivery methods and deepening our understanding of individual patient response patterns are critical to maximizing therapeutic outcomes. Continued research and clinical innovation are essential to refine INCS use across diverse CRS phenotypes, improve long-term adherence, and address gaps in care for underserved populations, including pediatric patients.
